# Structural, thermal and photo-physical data of azo-aromatic TEMPO derivatives before and after their grafting to polyolefins

**DOI:** 10.1016/j.dib.2015.12.047

**Published:** 2016-01-06

**Authors:** Francesca Cicogna, Ilaria Domenichelli, Serena Coiai, Fabio Bellina, Marco Lessi, Roberto Spiniello, Elisa Passaglia

**Affiliations:** a Istituto di Chimica dei Composti OrganoMetallici (ICCOM), Consiglio Nazionale delle Ricerche, SS Pisa, Via G. Moruzzi 1, 56124 Pisa, Italy; b Scuola Normale Superiore, Piazza dei Cavalieri 7, 56126 Pisa, Italy; c Dipartimento di Chimica e Chimica Industriale, Università degli Studi di Pisa, Via G. Moruzzi 13, 56124 Pisa, Italy

## Abstract

The data reported in this paper are complementary to the characterization of 4-(phenylazo)-benzoyl-2,2,6,6-tetramethylpiperidine-1-oxyl radical (AzO-TEMPO) and of the 4-(2-thienylazo)-benzoyl-2,2,6,6-tetramethylpiperidine-1-oxyl radical (ThiO-TEMPO) before and after their grafting to two polyethylene matrices (a copolymer ethylene/α-olefin (*co*-EO) and a high density polyethylene (HDPE)). Particularly the data reported in this paper confirm the structure (FT-IR analysis), the thermal (TGA and EPR) and the photo-physical (UV–vis) properties of the RO-TEMPO derivatives before and after their grafting. Herein, the FT-IR spectrum and TGA thermogram of ThiO-TEMPO were compared with those of AzO-TEMPO. Moreover, the superimposition of UV–vis spectra collected during the irradiation under 366 or 254 nm emitting lamp of AzO-TEMPO and ThiO-TEMPO in acetonitrile solution are reported. Finally, a complete DSC characterization of the functionalized POs is shown.

DOI of original article: 〈http://dx.doi.org/10.1016/j.polymer.2015.11.018〉 [1]

**Specifications Table**

**Table:** 

Subject area	*Chemistry*
More specific subject area	*Radical functionalization of polyolefins*
Type of data	*Spectra, graphs, thermograms*
How data was acquired	*FT-IR (Perkin Elmer Spectrum 100); TGA (Seiko EXSTAR 7200 TGA/DTA); EPR (Varian E112 spectrometer equipped with a Varian E257 temperature control unit); UV–vis (Perkin-Elmer Lambda 25 UV–vis Spectrometer); DSC (Perkin Elmer DSC-4000 equipped with a 3 stage cooler)*
Data format	*Analyzed*
Experimental factors	*Low molecular weight compounds were purified by column chromatography. Functionalized polymers were purified by dissolution and re-precipitation.*
Experimental features	*FT-IR spectra of low molecular weight compounds were collected from KBr pellets; FT-IR spectra of polymers were collected from films. TGA analyses were carried out on 10* *mg samples heating from 30 to 700* °*C at 10* °*C/min under nitrogen flow. EPR spectra were collected from a solid mixture of silica and TEMPO. UV–vis spectra of the low molecular weight compounds were recorded from acetonitrile solutions (about 5×10*^*-5*^ *M). UV–vis spectra of polymers were collected from films. DSC analysis were carried out on 10* *mg samples that were heated and cooled, choosing the appropriate temperature range, at 10* °*C/min under nitrogen flow.*
Data source location	*CNR ICCOM-SS Pisa (Italy)*
Data accessibility	*Data is available with this article.*

**Value of the data**

• Structural and thermal characterizations of the low molecular RO-TEMPO derivatives are published for the first time so are useful data to share with other researchers.

• The thermal properties (TGA and DSC) of functional polymers are important data to establish the service life of polymer materials.
• The superimposition of the UV–vis spectra, acquired during the irradiation of the RO-TEMPO in acetonitrile solution, gives a complete overview of the spectra between 200 and 600 nm that can be useful to choose the irradiation wavelength.

## 1 Data

The data related to the structural, thermal and photo-physical characterization of the low molecular weight TEMPO derivatives (RO-TEMPOs) as well as these of the functional POs bearing covalently linked the TEMPO derivatives (Cicogna et al., 2015, [1]) were reported in this paper. The aim is to share all data relative to this new TEMPO derivative and to compare these data with that of AzO-TEMPO (Nakatsuji et al. [2]) that was fully characterized in this paper. After the covalent grafting of both the RO-TEMPO derivatives to polyolefin (PO) by the nitroxide radical coupling (NRC) reaction (Cicogna et al., 2016, [1]), all characterizations were repeated with the aim to collect data about the structural, thermal and spectroscopic modification of the pristine PO derived from functionalization.

## 2 Experimental design, materials and methods

### 2.1 Materials

4-(Phenylazo)-benzoyl-2,2,6,6-tetramethylpiperidine-1-oxyl radical (AzO-TEMPO) and 4-(2-thienylazo)-benzoyl-2,2,6,6-tetramethylpiperidine-1-oxyl radical (ThiO-TEMPO) were synthesized as reported in Ref. [1]. High density polyethylene (HDPE) Lacqtene 2070MN60 produced by Arkema group having Mn=21,000 g/mol, Mw=86,000 g/mol, dispersity=4.10, density=0.96 g/cm^3^ and MFR=7 g/10 min (2.16 kg at 190 °C) and a random copolymer ethylene/α-olefin (*co*-EO) having Mn=50,000 g/mol, Mw=102,000 g/mol, dispersity=2.15, density=0.87 g/cm^3^ and MFR=4.7 g/10 (2.16 kg at 190 °C) min were used as polymer matrices. The functionalization of POs was carried out as previously reported [1]. The samples (*co*-EO)_170 and HPDE_190 were prepared by melt mixing the pristine polymers in an internal batch mixer (Brabender Plastograph OHG47055) with a chamber of 30 mL at 170 and 190 °C, respectively, for 20 min at 50 rpm, without the addition of peroxide and TEMPO derivatives.

### 2.2 Characterization

**Fig. 1:**
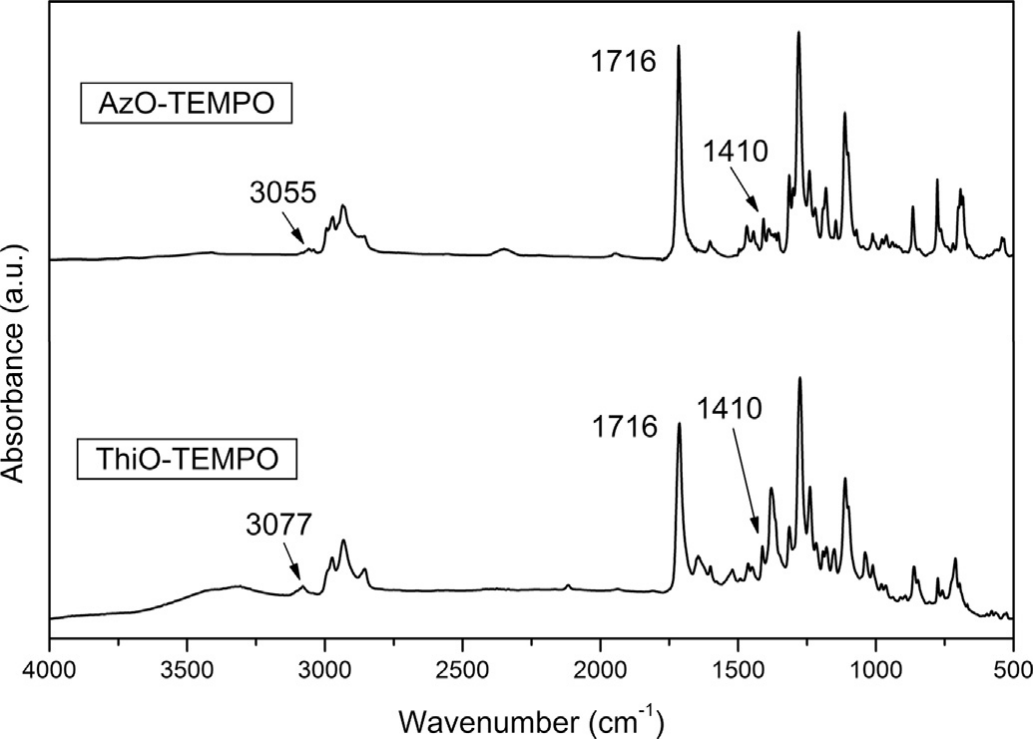
FT-IR spectrum of AzO-TEMPO and ThiO-TEMPO collected from KBr.

FT-IR spectra were recorded by the Fourier Transform Spectrometer Perkin Elmer Spectrum 100. Spectra of microcrystalline samples were obtained by potassium bromide pellet technique (Fig. 1).

**Fig. 2:**
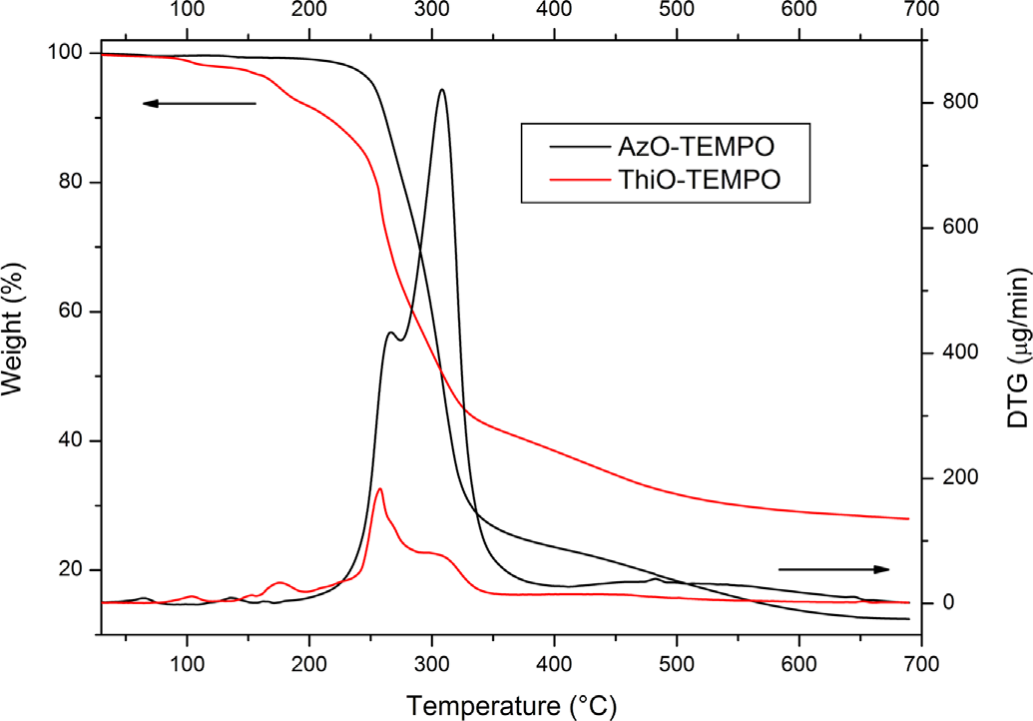
TGA thermograms and their first derivative of AzO-TEMPO and ThiO-TEMPO.

Thermo-gravimetric analyses (TGA) were carried out by using the instrument Seiko EXSTAR 7200 TGA/DTA. In a typical experiment, the sample (about 10 mg) was placed in an alumina sample pan and the run was carried out at 10 °C/min from 30 to 700 °C under nitrogen flow (Fig. 2, Fig. 4, Fig. 5).

**Fig. 3:**
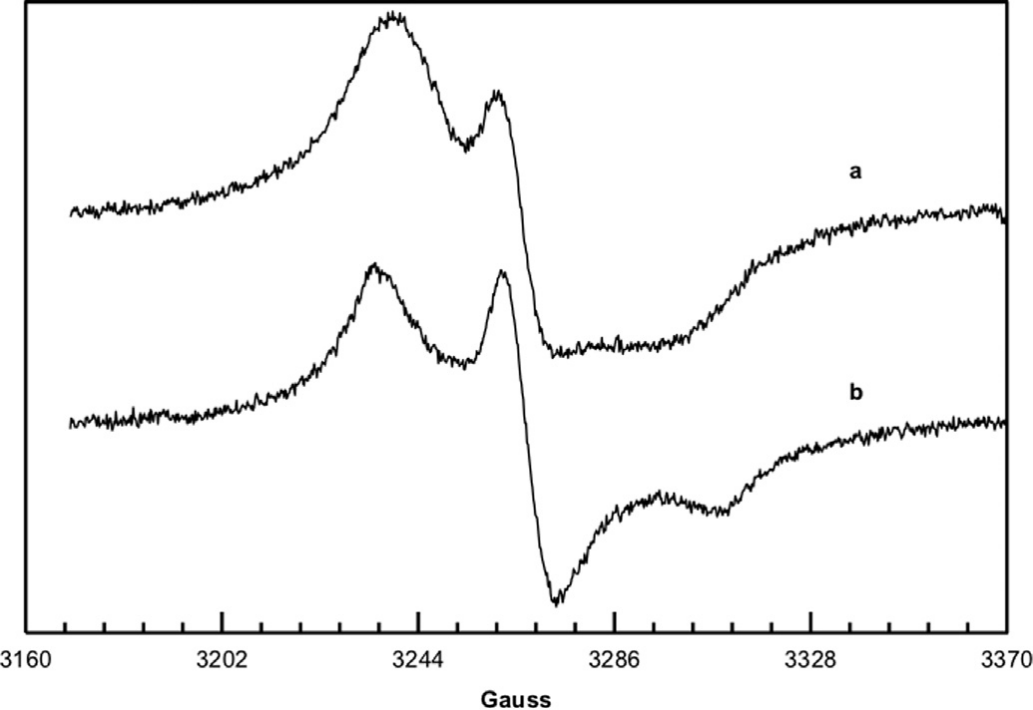
EPR spectrum of ThiO-TEMPO mixed with silica gel registered at room temperature before (a) and after heating at 190 °C for 10 min (b).

**Fig. 4:**
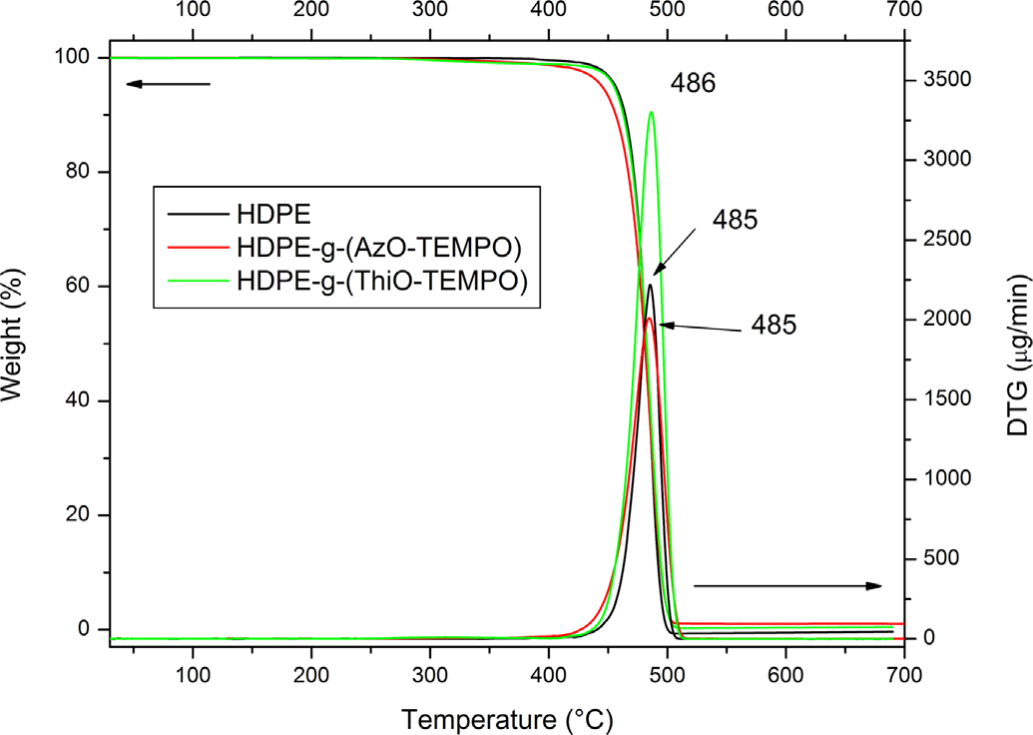
TGA thermograms and their first derivative of HDPE, HDPE-g-(AzO-TEMPO) and HDPE-g-(ThiO-TEMPO). The analyses were carried out under nitrogen flow at a heating rate of 10 °C/min.

**Fig. 5:**
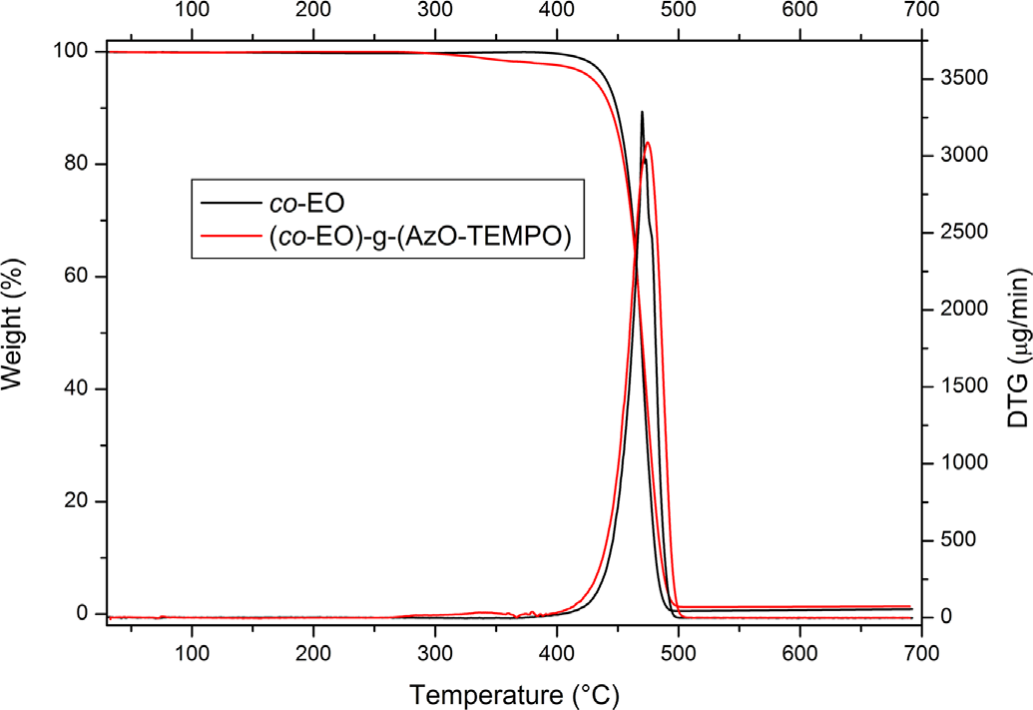
TGA thermograms and their first derivative *co*-EO and (*co*-EO)-g-(AzO-TEMPO). The analyses were carried out under nitrogen flow at a heating rate of 10 °C/min.

X-band EPR spectra were obtained with a Varian E112 spectrometer equipped with a Varian E257 temperature control unit. The EPR spectrometer was interfaced with an IPC 610/P566C industrial grade Advantech computer by means of a data acquisition system. This unit consists of an acquisition board capable of acquiring up to 500,000 12-bit samples per second. The sample was prepared by adding 0.1 mL of a solution of ThiO-TEMPO in dichloromethane (3.4×10^−4^ M) to 45 mg of silica gel, after the evaporation of the solvent, the solid sample was transferred into the quartz tube (3 mm internal diameter). Spectra were recorded at room temperature before and after the heating of the sample at 190 °C for 10 min (Fig. 3).

**Fig. 6:**
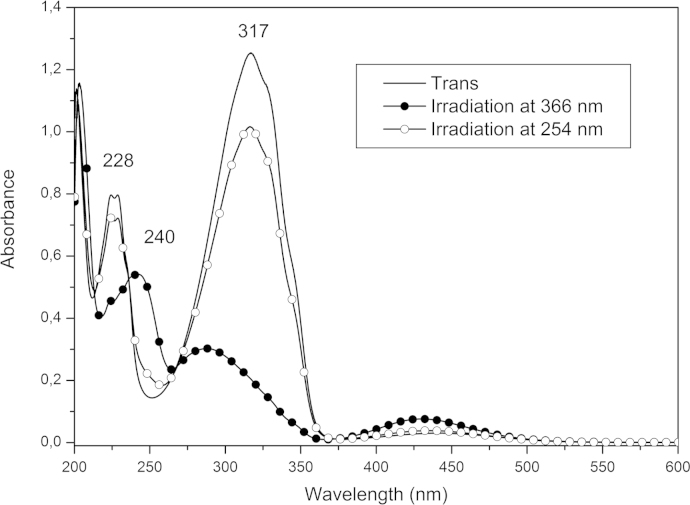
Absorption spectra of trans azobenzene (acetonitrile solution) and of azobenzene after irradiation at 366 nm and at 254 nm.

**Fig. 7:**
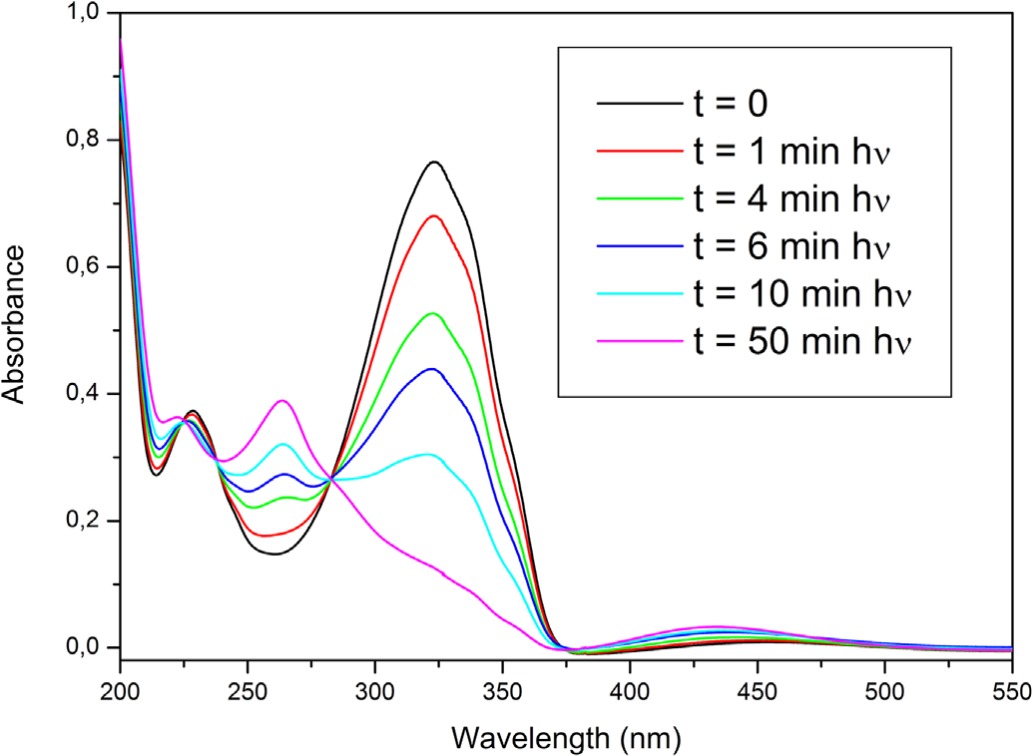
Absorption spectra of AzO-TEMPO (acetonitrile solution) collected after different irradiation times during irradiation at 366 nm.

**Fig. 8:**
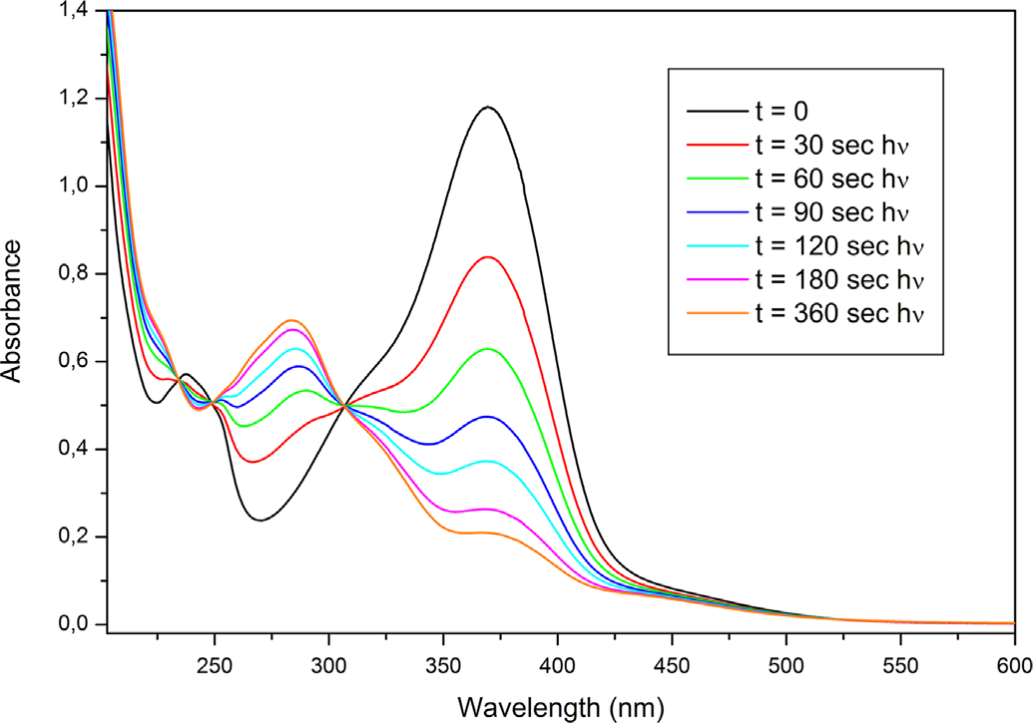
Absorption spectra of ThiO-TEMPO (acetonitrile solution) collected after different irradiation times during irradiation at 366 nm.

**Fig. 9:**
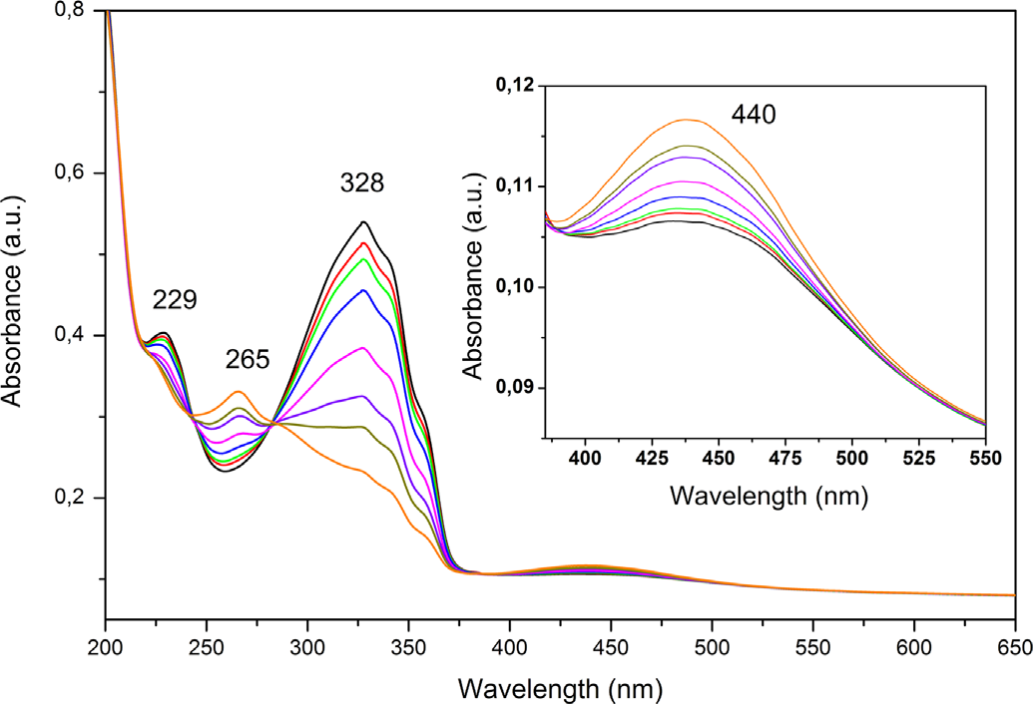
Absorption spectra of (*co*-EO)-g-(AzO-TEMPO) collected after different irradiation times during irradiation at 366 nm.

UV–vis absorption spectra were recorded at room temperature with a Perkin-Elmer Lambda 25 UV–vis Spectrometer. Acetonitrile solutions of AzO-TEMPO and ThiO-TEMPO (about 5×10^−5^ M) and films of functionalized polymers were analyzed. *co*-EO films were prepared by solution casting onto a quartz plate (Fig. 6, Fig. 7, Fig. 8, Fig. 9).

Photo-activation of azobenzene, AzO-TEMPO, ThiO-TEMPO and functionalized polymers was carried out with a Black Light equipment from Helios Italquartz Triwood 25/36 system equipped with 2 quartz glass lamps screened at 366 nm (6 W each) and with 2 quartz glass lamps screened at 254 nm (6 W each).

**Fig. 10:**
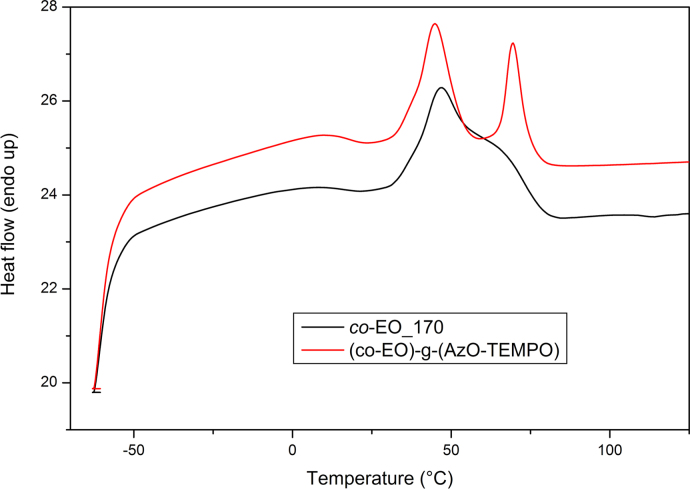
DSC curves of *co*-EO_170 and (*co*-EO)-g-(AzO-TEMPO), fist heating scan at 10 °C/min.

**Fig. 11:**
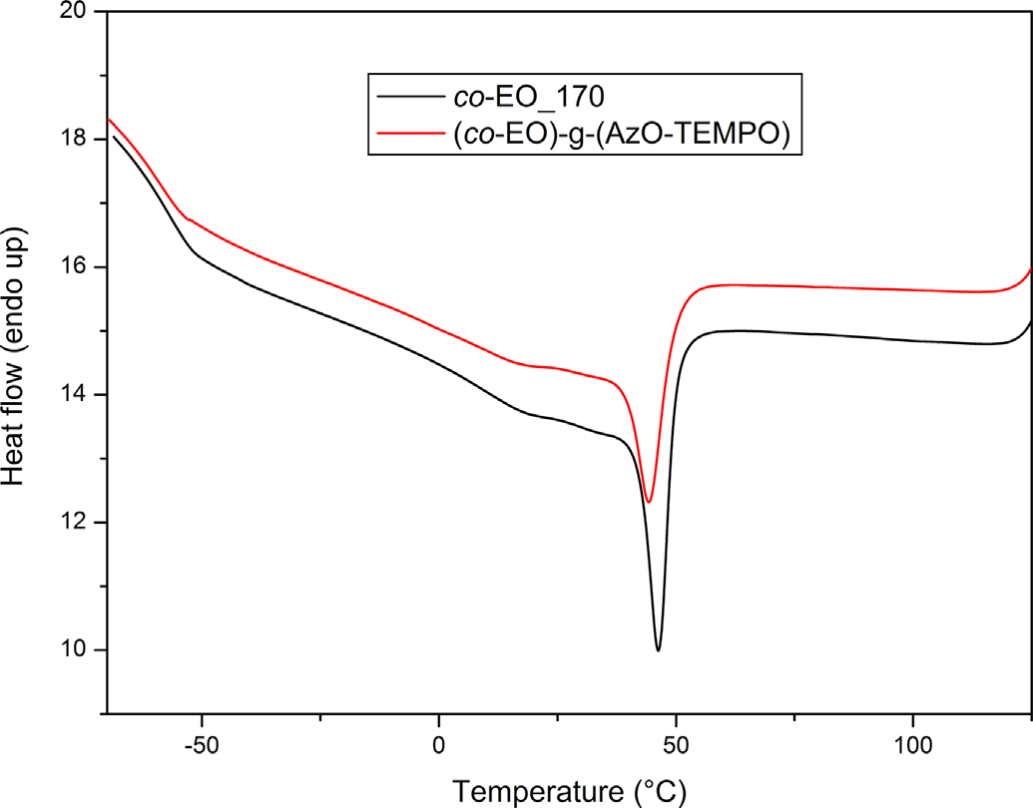
DSC curves of *co*-EO_170 and (*co*-EO)-g-(AzO-TEMPO), cooling scan at 10 °C/min.

**Fig. 12:**
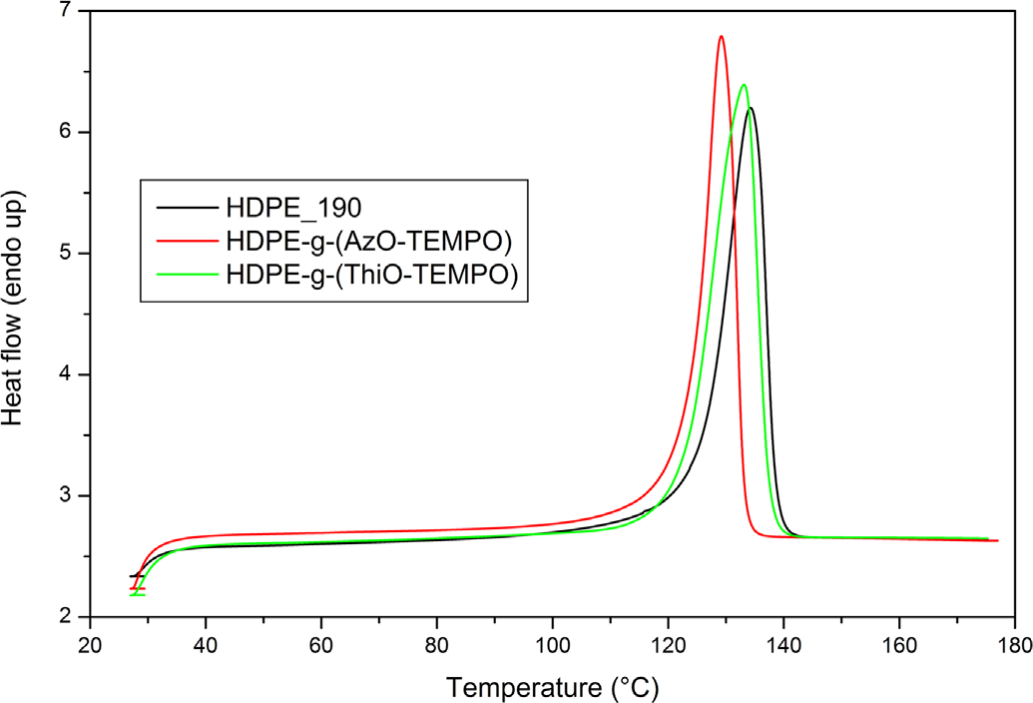
DSC curves of HDPE_190, HDPE-g-(AzO-TEMPO) and HDPE-g-(ThiO-TEMPO), second heating scan at 10 °C/min.

**Fig. 13:**
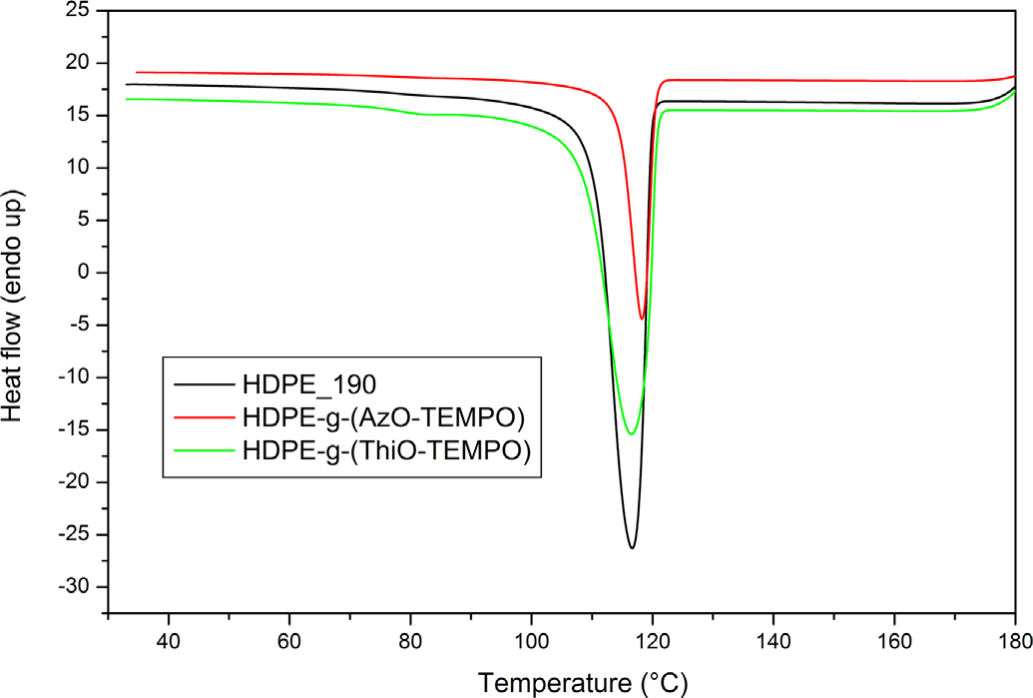
DSC curves of HDPE_190, HDPE-g-(AzO-TEMPO) and HDPE-g-(ThiO-TEMPO), cooling scan at 10 °C/min.

Differential Scanning Calorimetry (DSC) analysis was carried out using a Perkin-Elmer DSC-4000 differential scanning calorimeter thermal analyzer equipped with a 3 stage cooler able to reach −130 °C. Thermal scans were carried out on 10–15 mg samples under nitrogen atmosphere. Previously, the instrument was calibrated by using indium (m.p. 156.6 °C, Δ*H*=28.5 J/g) and zinc (m.p. 419.5 °C). HDPE samples were heated from 30 to 180 °C then cooled to 30 °C and heated again to 180 °C at a cooling/heating rate of 10 °C/min. *co*-EO samples were cooled to −60 °C, heated to 130 °C and cooled to 30 °C at a cooling/heating rate of 10 °C/min (Fig. 10, Fig. 11, Fig. 12, Fig. 13).
